# RNA-binding protein hnRNPK: a multifunctional regulator of skeletal muscle biology and disease

**DOI:** 10.1042/BST20250557

**Published:** 2026-05-21

**Authors:** Kejin Ren, Kaili Zhou, Yijia An, Xiaofang Cheng, Tiantian Meng, Cencen Li, Haixia Xu, Pengpeng Zhang, Yongjie Xu

**Affiliations:** 1 College of Life Science, Xinyang Normal University, Xinyang 464000, China; 2 Institute for Conservation and Utilization of Agro-Bioresources in Dabie Mountain, Xinyang Normal University, Xinyang 464000, China

**Keywords:** hnRNPK, muscle disease, regulation, skeletal muscle development

## Abstract

Heterogeneous nuclear ribonucleoprotein K (hnRNPK) is a highly conserved, multifunctional DNA/RNA-binding protein that regulates gene expression at both transcriptional and post-transcriptional levels. In skeletal muscle, hnRNPK is essential for development, regeneration, and homeostasis, influencing satellite cell activation, myoblast proliferation and differentiation, and myofiber maturation. Its dysregulation is linked to muscle atrophy, degenerative diseases, and impaired regeneration. This review summarizes current knowledge of hnRNPK’s molecular structure, subcellular localization and dynamics, and interactions with nucleic acids and proteins. We highlight its roles in myogenic differentiation, gene expression control, signaling pathway cross-talk, and skeletal muscle development. We also discuss the potential of hnRNPK as a diagnostic biomarker and therapeutic target in muscle disorders, and outline key directions for future research to resolve outstanding questions about its complex regulatory functions. Together, these insights provide a framework for advancing muscle biology and improving the management of muscle-related diseases.

## Introduction

Skeletal muscle, which accounts for 30%–50% of body mass, is essential for movement, energy metabolism, thermogenesis, and protein storage, thereby contributing critically to systemic homeostasis. It is composed of multinucleated myofibers, satellite cells (SCs), and supporting stromal and vascular components, and retains a remarkable capacity for regeneration throughout life. Understanding the molecular mechanisms that govern muscle development and repair is therefore a central goal in basic and translational research. Myogenesis proceeds through coordinated stages of proliferation, differentiation, fusion, and maturation, all of which are tightly regulated at the level of gene expression [1,2]. While transcriptional networks have been extensively characterized, the contributions of RNA-binding proteins (RBPs)—which control RNA metabolism and constitute roughly 2%–8% of eukaryotic proteins—remain comparatively underexplored in muscle biology [3].

RBPs, via conserved domains such as RRM, KH, and dsRBM, orchestrate RNA splicing, localization, stability, and translation. In skeletal muscle, they are critical for myogenic programs, shaping mRNA stability, translation, and alternative splicing and engaging in widespread interactions with noncoding RNAs (ncRNAs) [4,5]. For example, hnRNPD is up-regulated in activated SCs and binds AU-rich elements in 3′UTRs to control mRNA decay, thereby facilitating regeneration by regulating checkpoint transcripts and enhancing MEF2 synthesis [6]. GRSF1 supports muscle repair by binding G-rich RNAs and modulating GPX4-dependent mitochondrial reactive oxygen species (ROS) handling, a key step in myoblast activation [7]. PCBP1 cooperates with Ago2 to regulate maturation of muscle-specific miRNAs, thereby influencing gene expression during differentiation [8]. Other RBPs—including PTBP1, TDP-43, RBFOX2, and hnRNPA1—dynamically control alternative splicing events that underlie stage-specific transitions in muscle development and SC function [9–11]. Collectively, these findings establish RBPs as central post-transcriptional regulators of skeletal muscle biology.

Within this group, heterogeneous nuclear ribonucleoproteins (hnRNPs) represent major family of RBPs that shuttle between the nucleus and cytoplasm to regulate multiple aspects of RNA processing, including splicing, export, stability, and translation [12,13]. For many hnRNPs, including heterogeneous nuclear ribonucleoprotein K (hnRNPK), muscle-specific functions are only beginning to be defined. hnRNPK is emerging as an important regulator of muscle development and disease through its roles in transcriptional control, mRNA stability, translation, and ncRNA biology [14,15]. However, a comprehensive synthesis of its functions in skeletal muscle has been lacking. In this review, we examine hnRNPK’s regulatory roles in skeletal muscle, with a focus on its molecular mechanisms in myogenesis; its interactions with signaling pathways and other RBPs in controlling muscle function; and its dysregulation in muscle atrophy, degenerative disease, and impaired regeneration. We also evaluate hnRNPK’s potential as a diagnostic biomarker and therapeutic target. By integrating these dimensions, we underscore hnRNPK’s unique contributions to muscle biology, identify critical gaps in current knowledge, and outline priorities for future basic and translational research.

## RNA binding protein hnRNPK

### Molecular structure and functional properties

**Figure 1:**
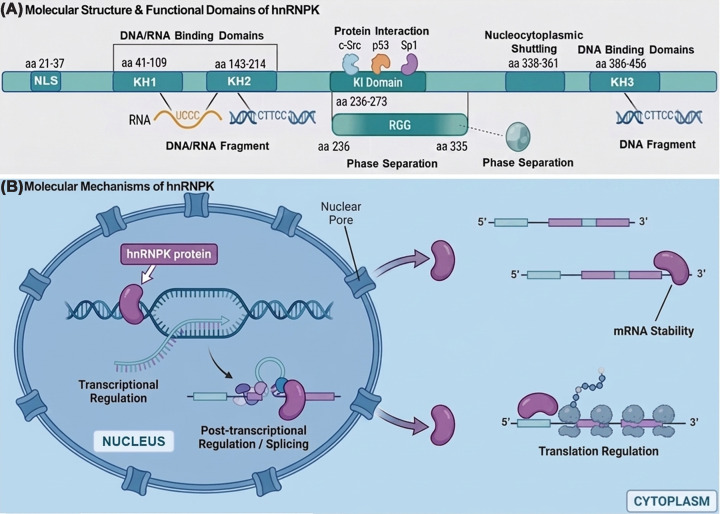
The molecular structure and molecular mechanisms of hnRNPK (**A**) Molecular structure and functional domains of hnRNPK. The linear schematic depicts the hnRNPK protein (464 amino acids) with its key functional domains annotated, including the NLS, three K-homology (KH) RNA/DNA-binding domains, a protein interaction domain (KI), an arginine-glycine-glycine (RGG) domain implicated in phase separation, and a K-protein nuclear shuttling (KNS) domain. Representative interactions are illustrated: the KH domains binding to specific RNA (UCCC) and DNA (CTTCC) motifs, and the KI domain interacting with partner proteins such as c-Src, p53, and Sp1. (**B**) Molecular mechanisms of hnRNPK: DNA/RNA binding and subcellular dynamics. The schematic delineates the diverse roles of hnRNPK in the regulation of gene expression across various cellular compartments within a eukaryotic cell. Within the nucleus, hnRNPK is involved in both transcriptional and post-transcriptional regulation. It directly binds to DNA, represented as a double helix, thereby modulating transcriptional activity. Simultaneously, hnRNPK interacts with nascent pre-mRNA transcripts, participating in splicing and other RNA-processing events. In the cytoplasm, hnRNPK associates with mature mRNA molecules, influencing their stability and interacting with the translation machinery, including ribosomes, to regulate protein synthesis.

hnRNPK is a 464-amino acid RNA/DNA-binding protein composed of four major functional modules (Figure 1A). It contains three KH domains (KH1–KH3) that recognize UC-rich RNA and the DNA motif “CTTCC,” with KH2 and KH3 contributing most of the binding affinity and regulatory activity [14,16]. The KI domain (aa 236–273) mediates interactions with transcription factors and signaling proteins. A C-terminal RGG-rich region (approximately aa 236–335) promotes liquid–liquid phase separation, enabling dynamic assembly of ribonucleoprotein condensates that regulate RNA metabolism [17]. hnRNPK also carries an N-terminal nuclear localization signal (NLS; aa 21–37) and a C-terminal KNS shuttling signal (aa 338–361), which together support nucleocytoplasmic trafficking and allow hnRNPK to coordinate nuclear transcription/splicing with cytoplasmic mRNA translation [18]. In addition, its low-complexity regions can switch between soluble, droplet-like, and hydrogel states in response to environmental cues, further expanding its functional versatility [19].

Functionally, hnRNPK integrates protein, RNA, and DNA interactions to regulate transcription, chromatin organization, pre-mRNA splicing, mRNA stability, and translation, thereby influencing proliferation, differentiation, apoptosis, DNA repair, stress responses, and immunity [15,20–22]. It also acts through noncoding RNAs, binding lncRNAs such as *XIST* and *NEAT1* to modulate gene expression in a cell-type-dependent manner [14,17]. In stem cells, hnRNPK contributes to fate control: for example, it cooperates with lineage regulators and RNA-processing factors to maintain pluripotency and guide differentiation programs, in part by shaping transcript splicing and chromatin states [21,23]. Finally, post-translational modifications (PTMs) fine-tune hnRNPK structure, interactions, and splicing activity, adding another regulatory layer [24,25]. Collectively, hnRNPK-centered RNA interactions represent a core mechanism of gene regulation across tissues and disease contexts and may provide actionable therapeutic entry points.

### Localization and nucleocytoplasmic dynamics

hnRNPK function depends strongly on its subcellular distribution and shuttling between the nucleus and cytoplasm (Figure 1B). External stimuli can rapidly shift this balance and alter hnRNPK outputs. For example, TRAIL treatment drives hnRNPK accumulation in the cytoplasm of H1299 cells and enhances its anti-apoptotic activity [26]. PTMs—particularly phosphorylation—are key determinants of localization and binding specificity. ERK1/2 phosphorylation at Ser284 (within the KI domain) and Ser353 (within the KNS region) impairs nuclear import and promotes cytoplasmic retention, where hnRNPK binds 3′UTRs of target mRNAs (e.g., *Tak1, Eif4e, Ucp2, c-Src*) to regulate translation [27,28]. Importantly, in C2C12 myoblasts, phosphorylation at Ser284/Ser353 is detected specifically during differentiation, suggesting muscle stage-dependent control of hnRNPK trafficking [29].

hnRNPK also regulates RNA localization of its bound RNAs. For instance, its binding to a SINEB1 element within the lncRNA *Malat1* promotes its nuclear retention; loss of this element shifts *Malat1* to the cytoplasm and perturbs TDP-43 localization and function [30]. Furthermore, under severe exogenous stress, such as Hepatitis C virus infection, hnRNPK relocalizes to lipid droplet-associated regions, colocalizes with viral components, and modulates viral production [31]. Together, these studies demonstrate that hnRNPK localization is not merely descriptive but mechanistic—linking signaling inputs to specific nuclear or cytoplasmic gene-regulatory programs. More broadly, this regulated shuttling is a common strategy used by other RBPs (e.g., SRSF1) and signaling regulators (e.g., SIRT1) to reprogram gene expression across development, differentiation, and stress [32,33].

### Synergistic with other proteins and RNAs

hnRNPK functions as a scaffold and regulatory hub, assembling protein–RNA complexes that support both normal physiology and disease-associated rewiring [34]. In cancer models, it cooperates with oncogenic and splicing factors—for example, interacting with MYC to promote tumorigenic programs [35] and partnering with SRSF1 to bias *CD44* pre-mRNA splicing toward oncogenic isoforms (the hnRNPK-SRSF1-CD44E axis) [36]. In normal biology, hnRNPK contributes to X chromosome inactivation by working with the PCGF3/5-PRC1 complex to enforce chromosome-wide silencing [37,38], and it can enhance translation in conjunction with PTBP1 through SINEUP lncRNAs [39]. hnRNPK may also collaborate with poly-C binding proteins (PCBP1/2) during oxidative stress responses, although the precise underlying mechanisms remain to be defined [40].

At the transcriptome level, hnRNPK participates in the formation of a highly cooperative and partially redundant regulatory network of the hnRNP family. More than half of alternative splicing events are co-regulated by multiple proteins in the hnRNP proteins. By engaging in complementary, binding-site-dependent interactions, these proteins enhance the robustness of splicing regulation and maintain splicing fidelity [41]. This cooperative mode has been verified at single-nucleotide resolution, confirming that hnRNP proteins exhibit precise, overlapping binding properties that fine-tune splicing networks [42]. Beyond protein interactions, hnRNPK interfaces with diverse ncRNA classes (lncRNAs, miRNAs, and piRNAs) to control transcriptional and post-transcriptional outputs and, in some contexts, chromatin accessibility [14]. For instance, in colorectal cancer, hnRNPK binds lncRNA *CRLM1* to increase promoter occupancy at metastasis-associated loci and amplify transcription [43], and it can reshape miRNA-regulated pathways (e.g., via miR-1207-5p and downstream *HO-1*) [44].

Overall, hnRNPK’s ability to integrate protein partners, RNA regulators, and chromatin-associated complexes explains its broad impact on transcription, splicing, and translation. While these synergistic networks have been most extensively mapped in cancer, the immense complexity of myogenesis and muscle repair strongly suggests that analogous, hnRNPK-centric regulatory hubs are equally critical for maintaining skeletal muscle homeostasis, warranting deeper investigation in neuromuscular models.

## Role of hnRNPK in muscle development

### Dynamic expression of hnRNPK in skeletal muscle

The expression of hnRNPK is tightly regulated during skeletal muscle development, ensuring the protein is available to properly orchestrate chromatin remodeling, transcription, splicing, and mRNA stability. Single-cell RNA sequencing of embryonic day 14 (E14) mouse skeletal muscle reveals high hnRNPK levels in SCs and muscle fibers [45]. In quiescent SCs, hnRNPK expression remains low but increases significantly upon cellular activation [46,47]. During embryogenesis, hnRNPK is strongly expressed to support myoblast proliferation and differentiation by modulating cell cycle regulators (e.g., *Ccna2*) and myogenic factors (e.g., *Myog*) [48–50]. Highlighting the evolutionary conservation of these pathways, hnRNPK has also emerged as a central regulator of growth-related genes in agricultural models, such as the black Muscovy duck [51]. After birth, however, hnRNPK levels naturally decline. This is consistent with the reduced proliferative demand of mature muscle and with extensive postnatal remodeling of alternative splicing programs, especially those controlling calcium handling [52,53]. These patterns indicate that hnRNPK-dependent RNA processing is particularly critical during embryonic development, where it maintains progenitor proliferation and prevents premature differentiation.

In pathological contexts, this expression pattern is frequently disrupted. For instance, hnRNPK is aberrantly up-regulated in aging skeletal muscle [54,55], suggesting its dysregulation contributes to age-related muscle decline rather than normal homeostatic maintenance. Supporting this, the overexpression of hnRNPK in adult mouse muscle actively impairs muscle integrity, likely by activating p53 and promoting aging phenotypes [53]. Conversely, in myocytes derived from ALS patients, hnRNPK is consistently down-regulated, implicating its loss in disease-associated transcriptional and post-ranscriptional defects [56]. Overall, hnRNPK is highly expressed during embryogenesis, reduced postnatally, and severely dysregulated in aging and ALS. This dynamic trajectory underscores its dual roles in development and disease, highlighting its potential as a stage-specific therapeutic target in muscle disorders.

### Function of hnRNPK in skeletal muscle development

**Figure 2:**
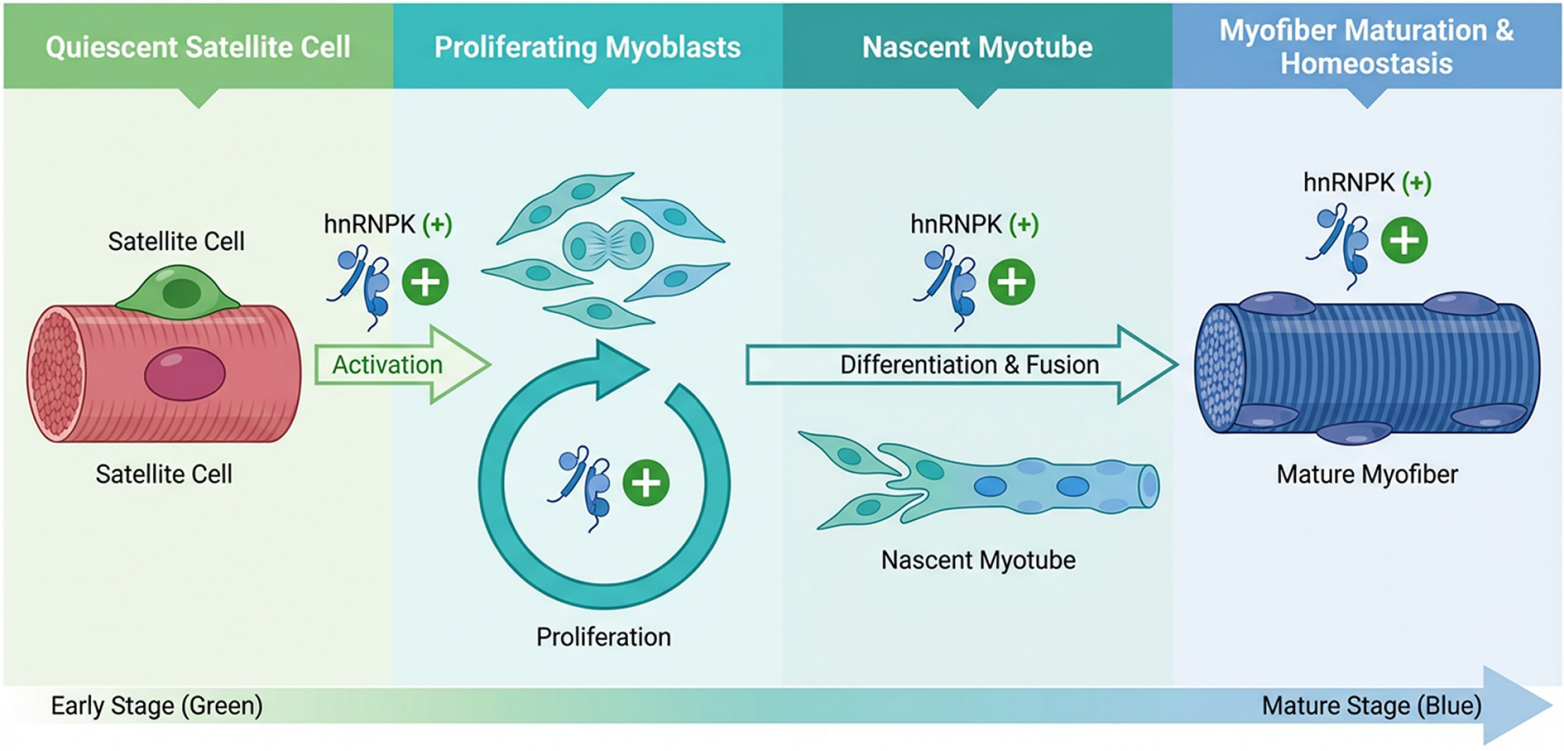
hnRNPK orchestrates skeletal muscle development and regeneration The diagram delineates the sequential stages of skeletal myogenesis, emphasizing the pivotal and stage-specific roles of hnRNPK. From left to right, the developmental progression is as follows: (i) Activation: quiescent SCs, resident on the surface of a mature myofiber, become activated. (ii) Proliferation: activated SCs give rise to a pool of proliferating myoblasts. (iii) Differentiation and Fusion: Myoblasts exit the cell cycle, differentiate, and fuse to form multinucleated myotubes. (iv) Maturation and Homeostasis: myotubes undergo further maturation into functional, striated muscle fibers. The plus signs (+) associated with hnRNPK icons at each transition point and its presence alongside the mature fiber collectively signify its positive regulatory role in driving and maintaining the entire myogenic program.

hnRNPK is essential for skeletal muscle development by coordinating transcription, RNA splicing, and translation, thereby regulating the balance between proliferation and differentiation (Figure 2). In proliferating C2C12 myoblasts, hnRNPK depletion causes G2/M phase arrest, reduces S-phase progression, and down-regulates key cell cycle regulators [50]. During myogenic differentiation, hnRNPK exerts context-dependent effects. On the one hand, it binds the lncRNA *Myoparr* to repress *Myog* transcription, preventing premature myoblast differentiation [57,58]. On the other hand, to initiate proper differentiation, it stabilizes *Cdkn1a* (*p21*) mRNA, which facilitates a controlled cell-cycle exit [47]. Consistent with these dual roles, the loss of hnRNPK in SCs triggers premature differentiation, yet its complete absence ultimately impairs proper myotube formation, resulting in structurally abnormal myotubes [50]. Thus, hnRNPK is indispensable for maintaining myoblast proliferation, ensuring the timely onset of differentiation, and preserving the overall balance between these states in SCs.

*In vivo* genetic studies further emphasize its non-redundant role. The deletion of hnRNPK in muscle precursor cells causes severe myogenic defects and perinatal lethality, highlighting its requirement for early myofiber formation [53]. In adult mice, myofiber-specific hnRNPK ablation leads to rapid body weight loss, kyphosis, premature death, profound muscle atrophy, and widespread transcriptional dysregulation. Conversely, hnRNPK overexpression also damages muscle tissue, causing fiber thinning and features of accelerated aging (Table 1) [53]. Together, these findings indicate that both insufficient and excessive hnRNPK are detrimental; precise hnRNPK dosage is required for normal muscle development and maintenance.

**Table 1:** Summary of the roles of hnRNPK in skeletal muscle development

No.	Models	Function	Regulatory mechanisms	Reference
1	C2C12 myoblasts	Prevent premature differentiation	hnRNPK combined with *lncRNA Myoparr* to inhibit myogenin transcription	[58]
2	C2C12 myoblasts	Maintain endoplasmic reticulum homeostasis	hnRNPK activate eIF2 α/AtF4 pathway and inhibit cytoplasmic aminoacyl tRNA synthase	[58]
3	C2C12 myoblasts and mouse	Promote myogenic differentiation	hnRNPK partners with hnRNPL to bind MyoD-induced seRNAs, activating target gene transcription	[60]
4	SCs and mice	Promote cell cycle arrest and limit self-renewal potential	hnRNPK binds to and regulates the mRNA of the target gene *Cdkn1a*	[47]
5	sheep myoblast	Inhibit myoblast proliferation while promoting differentiation	hnRNPK interacts with *LNC004268* to stabilize *CNOT2* mRNA	[61]
6	PSCs	determining the fate of PSCs	hnRNPK/miR-133a-3p/*UCP2* axis	[62]
7	AAV9-mediated overexpression mice	Accelerate muscle fiber aging	hnRNPK triggered ferroptosis via the p53/SLC7A11/GPX4 axis	[53]
8	ALS patients	Play a key role in early dendritic spine degeneration in ALS	Regulate alternative splicing of *SYNGAP1*	[67]

### Mechanisms of hnRNPK-mediated regulation of muscle genes

hnRNPK plays a crucial role in regulating muscle gene expression, particularly at the level of transcription. In C2C12 myoblasts, hnRNPK binds the lncRNA *Myoparr*, which shares a promoter with *Myog*, to control myogenin transcription and differentiation (Table 1) [57,58]. hnRNPK depletion increases *Myog* expression, accelerates differentiation, and results in abnormal myotubes, whereas hnRNPK overexpression suppresses *Myog*, delaying differentiation and impairing maturation [58]. Transcriptome profiling shows that knockdown of *Myoparr, Myog*, or *Hnrnpk* produces highly overlapping gene-expression changes, underscoring hnRNPK as a central node in this regulatory axis [59].

Beyond lncRNAs, hnRNPK cooperates with different transcription factors and nuclear proteins to strengthen muscle-specific transcription. During differentiation, it partners with hnRNPL to bind MyoD-induced super-enhancer RNAs (seRNAs), thereby activating the transcription of MyoD target genes and driving myogenic differentiation (Table 1) [60]. Furthermore, in SCs, hnRNPK deletion disrupts expression programs governing cellular activation and lineage commitment, confirming its importance in muscle development and regeneration [47].

hnRNPK also shapes muscle cell growth and differentiation by controlling mRNA stability and translation. It is strictly required for proper expression of cell cycle regulators and myogenic factors. For instance, Hitachi et al. found that hnRNPK inhibits myogenic differentiation by repressing *Myog* and modulating the eIF2α/Atf4 stress response pathway [58]. In sheep myoblasts, the *lncRNA LNC004268* recruits hnRNPK to stabilize *CNOT2* mRNA, promoting differentiation while limiting proliferation, thus revealing a lncRNA-dependent, hnRNPK-mediated mechanism (Table 1) [61]. In porcine skeletal muscle satellite cells (PSCs), hnRNPK, miR-133a-3p, and UCP2 form a regulatory circuit essential for differentiation [62]. Specifically, miR-133a-3p targets *UCP2* 3′UTR to repress its expression and enhance differentiation, while hnRNPK modulates *UCP2* expression at the translational level (Table 1). Together they finely tune *UCP2* expression and thereby dictate PSC fate. Additionally, in proliferating SCs, hnRNPK binds the 3′UTR of *Cdkn1a* mRNA to prevent its degradation and coordinate timely cell cycle exit (Table 1) [47]. These examples highlight hnRNPK as a vital integrator of signaling pathways that couple mRNA stability and translation to muscle development.

Furthermore, hnRNPK is a major regulator of alternative splicing, a process critical for muscle differentiation and function. Acting alongside other hnRNPs, it contributes to the regulation of more than half of all alternative splicing events, with functional redundancy and compensation among family members [41,63]. For example, hnRNPK affects the splicing of MRPL33, a gene linked to mitochondrial function and cancer progression [64]. While hnRNPK is a key player in this process, it operates within a complex network of splicing factors, including RBM24 and other hnRNP family members such as hnRNPA1, which are also known to modulate alternative splicing of muscle-specific genes to generate isoforms required for muscle structure and physiology [65,66]. Importantly, this splicing regulatory function extends to the broader neuromuscular unit. In ALS, a 3′UTR mutation in *SYNGAP1* creates an aberrant hnRNPK binding site, leading to altered splicing characterized by elevated α1 and reduced γ isoforms, accompanied by dendritic spine loss (Table 1) [67]. Blocking this hnRNPK binding with antisense oligonucleotides restores normal splicing and improves spine morphology, suggesting a promising therapeutic strategy. These findings underscore hnRNPK’s central role in alternative splicing in both muscle and the nervous system, highlighting its relevance to neuromuscular disease.

Finally, the recently described role of hnRNPK in mediating the nuclear import of SIRLOIN-containing RNAs opens exciting new avenues for understanding muscle biology and disease. This mechanism posits that hnRNPK acts as an adaptor, coupling specific ncRNAs to the nuclear import machinery, thereby controlling their subcellular localization and function [68,69]. In the context of skeletal muscle, this pathway could be crucial for delivering regulatory RNAs to the nucleus to modulate the transcription of myogenic genes (e.g., *Myog* and *MyoD* targets) or to maintain mRNA homeostasis. Disruption of SIRLOIN trafficking, whether through hnRNPK dysregulation or mutations in import receptors, could therefore directly contribute to the impaired regeneration, atrophy, and developmental defects observed in hnRNPK loss- and gain-of-function models [53]. Furthermore, given hnRNPK’s established role in cancer [36], the SIRLOIN pathway may represent a novel mechanism in tumorigenesis, potentially by controlling the nuclear availability of RNAs that regulate oncogenes or tumor suppressors. Intriguingly, this RNA-trafficking function may also have implications for genetic syndromes with shared clinical features. Kabuki syndrome (KMT2D/KMT6A), Au-Kline syndrome (HNRNPK), and Okamoto syndrome (HNRNPK) all involve dysregulation of transcriptional and epigenetic programs [70–72]. We hypothesize that hnRNPK-mediated SIRLOIN import could functionally link these pathways by directing specific non-coding RNAs to chromatin regulatory complexes, thereby influencing histone modification and gene expression. Defects in this process might therefore contribute to the overlapping developmental pathologies—such as skeletal abnormalities, intellectual disability, and growth retardation—seen in these disorders. Exploring the SIRLOIN interactome in muscle and other tissues could thus reveal unifying mechanisms of gene regulation and identify novel therapeutic targets for a spectrum of diseases.

### Comparison of hnRNPK with other hnRNP subfamily members in skeletal muscle

hnRNPK is distinguished within the hnRNP family by its “multidimensional cross-nucleocytoplasmic regulation.” It combines dual DNA/RNA-binding capacity with nucleocytoplasmic shuttling and concurrently participates in chromatin remodeling, transcriptional regulation, RNA splicing, mRNA stability, and translational control. Furthermore, its expression spans the entire course of skeletal muscle development, supporting multiple stages and processes. By contrast, many other hnRNPs exhibit more stage-restricted and functionally focused roles, often confined to a single post-transcriptional layer. For example, hnRNPL acts primarily at the onset of myocyte differentiation: by binding MyoD-induced seRNAs, it specifically activates transcription of muscle-specific genes and serves as a key transcriptional coactivator for differentiation initiation [60]. Similarly, hnRNPD plays a highly focused role downstream of METTL3; although primarily demonstrated in cardiomyocytes, it recognizes METTL3-dependent m^6^A sites on *TFEB* mRNA to repress its translation, thereby modulating autophagy and apoptosis in striated muscle [73]. hnRNPA1 and hnRNPA2B1, on the other hand, mainly regulate RNA metabolism, especially pre-mRNA alternative splicing [66]. Under basal conditions, they regulate muscle-related splicing in the nucleus and promote stress granule assembly via their C-terminal prion-like domains to protect mRNAs. Disease-associated mutations disrupt their nuclear localization, leading to cytoplasmic inclusions in muscle fibers, muscle atrophy, and direct links to multisystem proteinopathy and ALS.

In summary, hnRNPK acts as a central regulator of muscle gene expression, integrating transcriptional control, alternative splicing, mRNA stability, and translation, and interacting with lncRNAs, miRNAs, transcription factors, and other hnRNPs. Its tightly regulated expression and dosage-sensitive functions are crucial for muscle differentiation, development, regeneration, and homeostasis. Consequently, the dysregulation of hnRNPK contributes to muscle wasting, aging, and neuromuscular disease, making it a highly attractive therapeutic target for a broad spectrum of skeletal muscle disorders.

## hnRNPK and muscle-related diseases

### Mechanism of hnRNPK in muscular atrophy

**Figure 3:**
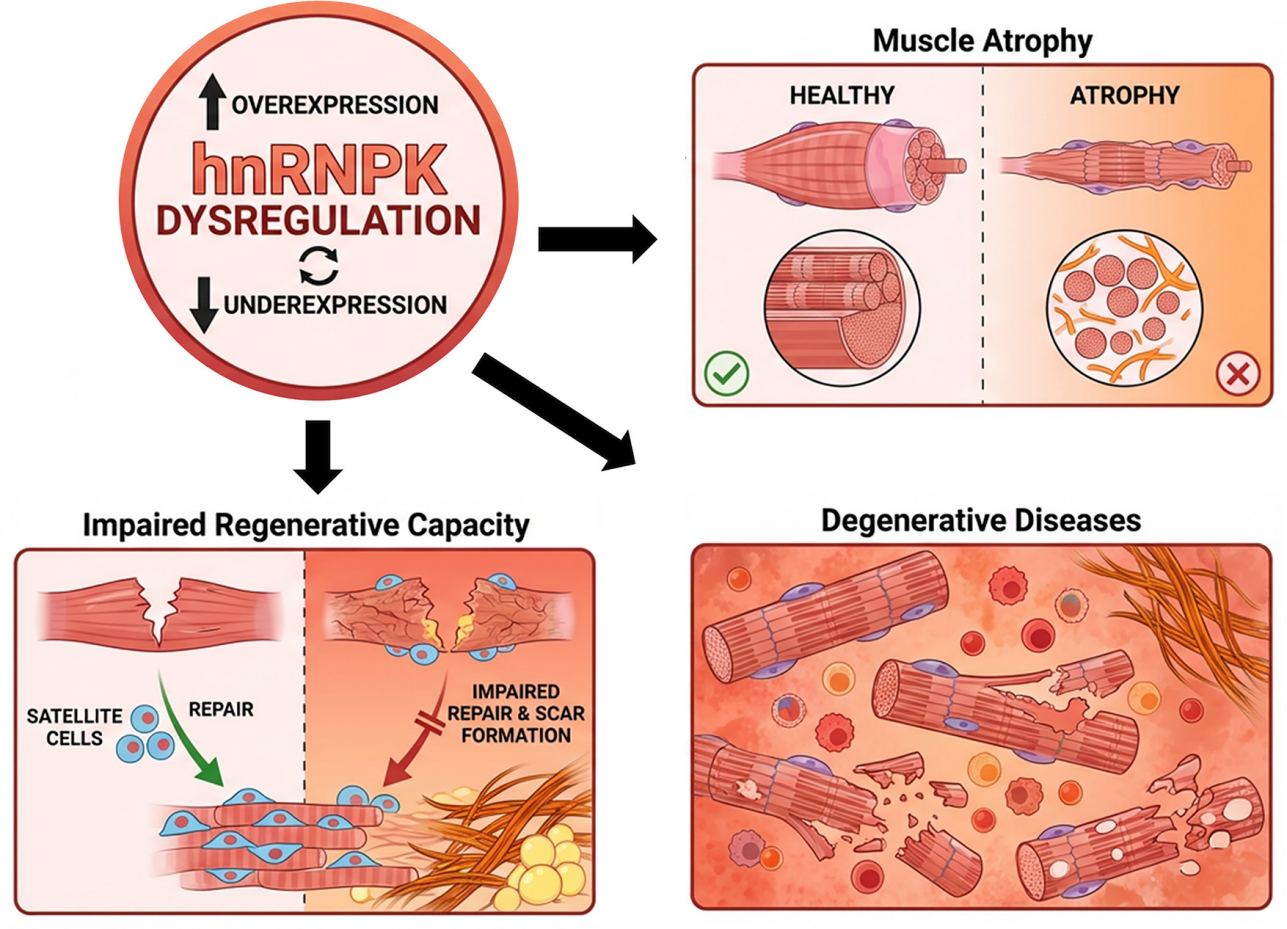
Pathological outcomes of hnRNPK dysregulation in skeletal muscle This hub-and-spoke diagram illustrates the central role of hnRNPK dysregulation (aberrant expression or function) as a pivotal node in driving diverse skeletal muscle pathologies. Three primary pathological axes emanate from this central dysfunction: (i) Muscle atrophy, characterized by the progressive loss of muscle mass and reduction in myofiber cross-sectional area. (ii) Degenerative diseases: Encompasses conditions marked by myofiber disorganization, degeneration, and the pathological deposition of fibrotic or inflammatory tissue. (iii) Impaired regenerative capacity: reflects a failure of the normal muscle repair program, where SC activation and differentiation are compromised, leading to ineffective regeneration and scar formation following injury. Collectively, these interconnected pathways highlight the multifaceted consequences of hnRNPK imbalance on muscle structure, function, and repair.

Muscle atrophy, characterized by loss of muscle mass and strength, arises from diverse conditions. Recent work identifies hnRNPK as a key regulator of myofiber survival and function, likely acting through multiple signaling and gene regulatory pathways [50,58]. In a myofiber-specific inducible knockout model (*Hnrnpk* aKO), hnRNPK deficiency causes widespread transcriptional dysregulation, increased apoptosis, and profound muscle atrophy (Figure 3) [53]. RNA-seq analysis shows up-regulation of genes linked to apoptosis, atrophy, and proteolysis, and down-regulation of genes involved in contraction and extracellular matrix organization. However, the precise molecular networks through which hnRNPK controls muscle homeostasis remain incompletely defined.

Although the direct mechanisms of hnRNPK in specific genetic dystrophies are still emerging, evidence from related pathologies provides valuable clues. For example, in oculopharyngeal muscular dystrophy, driven primarily by polyalanine expansions in PABPN1, mitochondrial dysfunction and PABPN1 mislocalization contribute to pathology [74]. Given hnRNPK's roles in transcription and signaling, it may similarly influence atrophy by modulating mitochondrial gene expression. Insights from other hnRNPs further support this hypothesis: the loss of hnRNPU in muscle induces immune cell infiltration, NF-κB activation, and atrophy [75]. Because NF-κB is a well-known driver of catabolic programs and protein degradation in atrophic muscle [76], it is plausible that hnRNPK dysfunction triggers similar inflammatory and proteolytic cascades. Thus, deciphering these overlapping networks makes targeting hnRNPK a promising potential therapeutic strategy for muscle atrophy.

Furthermore, hnRNPK may function by modulating oxidative stress and mitochondrial function, which are key determinants of muscle atrophy. For instance, in cancer cells, hnRNPK regulates the alternative splicing of the mitochondrial ribosomal protein MRPL33, thereby altering the expression of mitochondrial function-related genes, leading to ROS accumulation, reduced ATP production, and impaired mitochondrial translation [64]. In skeletal muscle, overexpression of hnRNPK exacerbates oxidative stress and activates the ferroptosis pathway by inhibiting the SLC7A11/GPX4 axis; simultaneously, it down-regulates the expression of oxidative phosphorylation-related genes, thus disrupting muscle homeostasis and promoting atrophy [53]. Conversely, another skeletal muscle study further demonstrated that hnRNPK can also cooperate with the methyltransferase SETD3 to regulate mRNA alternative splicing. By inducing exon skipping of the FNIP1 gene, hnRNPK promotes the nuclear translocation of the transcription factor TFEB, which in turn regulates mitochondrial biogenesis and maintains muscle mass [25]. Because antioxidants can alleviate muscle loss in multiple atrophy models [77], this strongly supports the importance of the hnRNPK-mediated oxidative stress pathway. Overall, hnRNPK appears to integrate inflammatory signaling, proteolytic pathways, and oxidative stress in muscle atrophy. Clarifying these interactions will be critical for designing targeted interventions.

### hnRNPK and muscle degenerative diseases

Muscle degenerative disorders, including muscular dystrophies and sarcopenia, cause progressive weakness, atrophy, and loss of mobility, profoundly affecting quality of life through pain, fatigue, and mood disturbances [78]. hnRNPK, a multifunctional transcriptional and post-transcriptional regulator, has emerged as a contributor to these diseases, in part through its effects on muscle-bone cross-talk and skeletal integrity [79]. In ALS, hnRNPK exerts compartment-specific effects. Nuclear hnRNPK can mitigate *C9orf72*-related RNA toxicity, whereas cytoplasmic mislocalization is linked to stress granule abnormalities and neuronal loss (Figure 3) [80,81]. In addition, aberrant hnRNPK binding to a mutant *SYNGAP1* 3′UTR drives mis-splicing; antisense oligonucleotides that block this interaction correct splicing defects and represent a promising therapeutic approach [67]. These findings are consistent with the consensus in the field that dysregulated RNA metabolism—including altered translation and dynamic changes in stress granules—represents a core pathogenic mechanism of ALS [82]. Furthermore, they highlight hnRNPK as a critical node in this pathology, laying a strong theoretical foundation for therapeutic interventions aimed at correcting hnRNPK-mediated RNA metabolism.

Mechanistic links between hnRNPK and degenerative muscle disease are further supported by recent atrophy studies implicating mitochondrial dysfunction, immune-mediated regulation of muscle mass, and microRNA-controlled catabolic pathways. A deeper understanding of how hnRNPK intersects with these processes is likely to inform the development of novel, effective therapies for ALS, sarcopenia, and related neuromuscular conditions.

### hnRNPK in muscular dystrophy

Muscular dystrophies are a group of hereditary muscle disorders driven by genetic mutations that lead to progressive muscle weakness, structural degeneration, and chronic inflammation. hnRNPK contributes to their pathophysiology through its roles in metabolism, autophagy/mitophagy, and RNA processing. For instance, it regulates lipid metabolism and mitochondrial quality control; in doxorubicin-induced cardiotoxicity models, hnRNPK overexpression enhances mitophagy via the PINK1/Parkin pathway and ameliorates lipid accumulation [83]. Given the high conservation of these metabolic networks, similar hnRNPK-mediated mechanisms are likely relevant in skeletal muscular dystrophies, which are also heavily characterized by impaired energy metabolism and mitochondrial dysfunction. Furthermore, hnRNPK modulates autophagy through the Beclin1 pathway [84], which is essential for maintaining muscle fiber integrity and is frequently disrupted in dystrophic wasting. In addition, its role in alternative splicing may influence the expression of structural proteins, such as dystrophin, whose absence underlies Duchenne muscular dystrophy [36]. Taken together, hnRNPK’s regulation of metabolic homeostasis, autophagy/mitophagy, and splicing highlights it as a promising therapeutic target in muscular dystrophies.

### hnRNPK and muscle regeneration disorders

Muscle regeneration depends on SCs, which orchestrate repair and growth after injury. In disorders such as muscular dystrophies, SC regenerative capacity is compromised, leading to defective healing and chronic inflammation [85]. hnRNPK is a key regulator of this process, controlling SC proliferation and differentiation through its effects on gene expression [50,58,60]. Using *Hnrnpk*^flox/flox^ and Pax7 CreER mice, we created SCs-specific knockout mice (*Hnrnpk* pKO) and found that hnRNPK loss reduces SCs proliferation and prematurely triggers differentiation. Consequently, in *in vivo* muscle injury models, hnRNPK deficiency impairs expansion of the SCs pool and accelerates differentiation, resulting in defective new myofiber formation and poor regeneration (Figure 3). Mechanistically, hnRNPK binds the 3′UTR of *Cdkn1a* mRNA to regulate its stability during SC proliferation and differentiation, thereby fine-tuning cell cycle exit [47]. Dissecting how hnRNPK and its protein and RNA partners coordinate SC behavior will be crucial for identifying therapeutic targets to enhance muscle repair and treat regeneration disorders.

### Potential of hnRNPK as a biomarker in muscle disease

Robust biomarkers are essential for diagnosing muscle diseases, monitoring progression, and evaluating therapeutic responses. They can be broadly divided into disease-unspecific, pathway-specific, and disease-specific markers [86]. Recent advances in skeletal muscle proteomics have revealed numerous fiber-associated proteins that are altered in neuromuscular disorders [87]. hnRNPK has emerged as a highly promising biomarker candidate because it lies at the intersection of transcription, splicing, and mRNA stability—processes commonly disrupted in myopathies. It is strictly required for myoblast proliferation and differentiation, and also contributes to systemic processes like cancer biology and bone homeostasis [50,79]. Importantly, hnRNPK expression is dynamically regulated across disease states: it is down-regulated in ALS patient-derived iPSCs, up-regulated in aging muscle, and altered in various atrophy and dystrophy models [54–56]. These disease- and stage-specific patterns, together with mechanistic links between hnRNPK modifications, interaction partners, and muscle pathology [24,50], strongly support its potential diagnostic value. Future clinical translation should focus on: (i) correlating serum or plasma hnRNPK levels with the severity and progression of muscle atrophy and dystrophy in human cohorts; and (ii) quantitatively assessing hnRNPK expression and subcellular localization in muscle biopsies (e.g., by immunohistochemistry or imaging-based assays). Defining pathological thresholds and localization signatures of hnRNPK could establish it as a clinically useful biomarker and aid in stratifying patients for hnRNPK-targeted therapies.

## Conclusion and future prospects

hnRNPK is a multifunctional DNA/RNA-binding protein that occupies a central position in skeletal muscle development, regeneration, and disease. As muscle research shifts from single-gene studies to systems-level analyses, hnRNPK has emerged as a key regulatory hub and an attractive focus for future investigation.

Emerging technologies will greatly refine our understanding of how hnRNPK operates within these complex networks. For instance, single-cell multi-omics and spatial transcriptomics can map its dynamic expression across distinct muscle cell populations and developmental stages, revealing precise, cell-type-specific regulatory circuits [45,88]. Simultaneously, resolving hnRNPK’s highly context-dependent actions will require advanced mechanistic tools. Defining the spatiotemporal specificity of its interactions with RNAs (e.g., *Myoparr*) and proteins (e.g., CTCF) can be achieved through high-resolution approaches such as proximity ligation assays, *in vivo* cross-linking, and cryo-electron microscopy, which can capture hnRNPK conformational changes during RNA binding [89]. Furthermore, gene editing tools such as CRISPR–Cas9, especially when combined with conditional knockout models, will enable the precise dissection of hnRNPK’s interactions with major signaling pathways, clarifying its contributions to muscle homeostasis.

Ultimately, translating these biological insights into therapies will demand solutions to delivery and safety challenges. This includes the efficient targeting of AAV vectors to SCs and minimizing the off-target effects of hnRNPK-directed interventions. Overall, hnRNPK is a multifaceted regulator at the intersection of muscle development, metabolism, and pathology. Fully elucidating its functions will deepen our understanding of muscle biology and accelerate the development of targeted therapies for a broad spectrum of muscle disorders.

## Perspectives

- Skeletal muscle biology and disease research is critical for addressing global health challenges like sarcopenia and ALS, and hnRNPK fills a key gap in understanding post-transcriptional regulation of myogenesis and muscle homeostasis.
- Current research establishes hnRNPK as a multifunctional regulator—governing satellite cell balance, muscle gene expression, and disease pathways—with its activity dependent on structure, localization, and expression dose.
- Future work should leverage high-resolution techniques to clarify hnRNPK’s context-specific mechanisms and validate its clinical potential as a biomarker or therapeutic target for muscle disorders.

## Open Access

Open access for this article is provided via our Subscribe to Open programme, made possible through the support of our subscribing institutions. Learn more here: https://portlandpress.com/pages/open_access_options_and_prices#conditional.

## Abbreviations

AI: artificial intelligence
hnRNPK: heterogeneous nuclear ribonucleoprotein K
hnRNPs: heterogeneous nuclear ribonucleoproteins
KH: K-homology
KNS: K-protein nuclear shuttling
NLS: nuclear localization signal
ncRNAs: noncoding RNAs
PTMs: post-translational modifications
PSCs: porcine skeletal muscle satellite cells
RBPs: RNA-binding proteins
ROS: reactive oxygen species
SCs: satellite cells
seRNAs: super-enhancer RNAs

## References

[1] Feng L.T., Chen Z.N. and Bian H. (2024) Skeletal muscle: molecular structure, myogenesis, biological functions, and diseases. MedComm. (2020) 5, e649 10.1002/mco2.649 38988494 PMC11234433

[2] Jiang H., Liu B., Lin J., Xue T., Han Y., Lu C. et al. (2024) MuSCs and IPCs: roles in skeletal muscle homeostasis, aging and injury. Cell. Mol. Life Sci. 81, 67 10.1007/s00018-023-05096-w 38289345 PMC10828015

[3] Avila-Lopez P. and Lauberth S.M. (2024) Exploring new roles for RNA-binding proteins in epigenetic and gene regulation. Curr. Opin. Genet. Dev. 84, 102136 10.1016/j.gde.2023.102136 38128453 PMC11245729

[4] Wu J., Niu L., Yang K., Xu J., Zhang D., Ling J. et al. (2024) The role and mechanism of RNA-binding proteins in bone metabolism and osteoporosis. Ageing Res. Rev. 96, 102234 10.1016/j.arr.2024.102234 38367813

[5] Wang X., Li J., Zhang C., Guan X., Li X., Jia W. et al. (2025) Old players and new insights: unraveling the role of RNA-binding proteins in brain tumors. Theranostics 15, 5238–5257 10.7150/thno.113312 40303323 PMC12036871

[6] Chenette D.M., Cadwallader A.B., Antwine T.L., Larkin L.C., Wang J., Olwin B.B. et al. (2016) Targeted mRNA decay by RNA binding protein AUF1 regulates adult muscle stem cell fate, promoting skeletal muscle integrity. Cell Rep. 16, 1379–1390 10.1016/j.celrep.2016.06.095 27452471 PMC5323095

[7] Yin W., Yang L., Kong D., Nie Y., Liang Y. and Teng C.B. (2019) Guanine-rich RNA binding protein GRSF1 inhibits myoblast differentiation through repressing mitochondrial ROS production. Exp. Cell. Res. 381, 139–149 10.1016/j.yexcr.2019.05.004 31085189

[8] Espinoza-Lewis R.A., Yang Q., Liu J., Huang Z.P., Hu X., Chen D. et al. (2017) Poly(C)-binding protein 1 (Pcbp1) regulates skeletal muscle differentiation by modulating microRNA processing in myoblasts. J. Biol. Chem. 292, 9540–9550 10.1074/jbc.M116.773671 28381556 PMC5465481

[9] Susnjar U., Skrabar N., Brown A.L., Abbassi Y., Phatnani H., Consortium N.A. et al. (2022) Cell environment shapes TDP-43 function with implications in neuronal and muscle disease. Commun. Biol. 5, 314 10.1038/s42003-022-03253-8 35383280 PMC8983780

[10] Singh R.K., Xia Z., Bland C.S., Kalsotra A., Scavuzzo M.A., Curk T. et al. (2014) Rbfox2-coordinated alternative splicing of Mef2d and Rock2 controls myoblast fusion during myogenesis. Mol. Cell 55, 592–603 10.1016/j.molcel.2014.06.035 25087874 PMC4142074

[11] Li Z., Wei H., Hu D., Li X., Guo Y., Ding X. et al. (2023) Research progress on the structural and functional roles of hnRNPs in muscle development. Biomolecules 13, 1434 10.3390/biom13101434 37892116 PMC10604023

[12] Li B., Wen M., Gao F., Wang Y., Wei G. and Duan Y. (2024) Regulation of HNRNP family by post-translational modifications in cancer. Cell Death Discov. 10, 427 10.1038/s41420-024-02198-7 39366930 PMC11452504

[13] Maceratessi S. and Sampaio N.G. (2024) hnRNPs in antiviral innate immunity. Immunology 173, 425–441 10.1111/imm.13846 39111743

[14] Xu Y., Wu W., Han Q., Wang Y., Li C., Zhang P. et al. (2019) New insights into the interplay between non-coding RNAs and RNA-binding protein HnRNPK in regulating cellular functions. Cells 8, 62 10.3390/cells8010062 30658384 PMC6357021

[15] Aguilar-Garrido P., Velasco-Estevez M., Navarro-Aguadero M.A., Otero-Sobrino A., Ibanez-Navarro M., Marugal M.A. et al. (2025) The tumor suppressor HNRNPK induces p53-dependent nucleolar stress to drive ribosomopathies. J. Clin. Invest. 135, e183697 10.1172/JCI183697 40338663 PMC12165811

[16] Wu W.Q., Zhang X., Bai D., Shan S.W. and Guo L.J. (2022) Mechanistic insights into poly(C)-binding protein hnRNP K resolving i-motif DNA secondary structures. J. Biol. Chem. 298, 102670 10.1016/j.jbc.2022.102670 36334628 PMC9709238

[17] Ding M., Wang D., Chen H., Kesner B., Grimm N.B., Weissbein U. et al. (2025) A biophysical basis for the spreading behavior and limited diffusion of Xist. Cell 188, 978–997, 10.1016/j.cell.2024.12.004 39824183 PMC11863002

[18] Habelhah H., Shah K., Huang L., Ostareck-Lederer A., Burlingame A.L., Shokat K.M. et al. (2001) ERK phosphorylation drives cytoplasmic accumulation of hnRNP-K and inhibition of mRNA translation. Nat. Cell Biol. 3, 325–330 10.1038/35060131 11231586

[19] Le Q.D., Lewis A., Dix-Matthews A., Ringler P., Duff A., Whitten A.E. et al. (2025) Structural characteristics and properties of the RNA-binding protein hnRNPK at multiple physical states. Int. J. Mol. Sci. 26, 1356 10.3390/ijms26031356 39941124 PMC11818384

[20] Chen Y., Wu J., Zhang S., Gao W., Liao Z., Zhou T. et al. (2022) Hnrnpk maintains chondrocytes survival and function during growth plate development via regulating Hif1α–glycolysis axis. Cell Death Dis. 13, 803 10.1038/s41419-022-05239-0 36127325 PMC9489716

[21] Huang Y., Liu Y., Pu M., Zhang Y., Cao Q., Li S. et al. (2024) SOX2 interacts with hnRNPK to modulate alternative splicing in mouse embryonic stem cells. Cell Biosci. 14, 102 10.1186/s13578-024-01284-8 39160617 PMC11331657

[22] Li J., Chen Y., Xu X., Jones J., Tiwari M., Ling J. et al. (2019) HNRNPK maintains epidermal progenitor function through transcription of proliferation genes and degrading differentiation promoting mRNAs. Nat. Commun. 10, 4198 10.1038/s41467-019-12238-x 31519929 PMC6744489

[23] Tang S., Xie Z., Wang P., Li J., Wang S., Liu W. et al. (2019) LncRNA-OG promotes the osteogenic differentiation of bone marrow-derived mesenchymal stem cells under the regulation of hnRNPK. Stem Cells 37, 270–283 10.1002/stem.2937 30372559 PMC7379496

[24] Xu Y., Wu W., Han Q., Wang Y., Li C., Zhang P. et al. (2019) Post-translational modification control of RNA-binding protein hnRNPK function. Open Biol. 9, 180239 10.1098/rsob.180239 30836866 PMC6451366

[25] Kong Y.Y., Shu W.J., Wang S., Yin Z.H., Duan H., Li K. et al. (2024) The methyltransferase SETD3 regulates mRNA alternative splicing through interacting with hnRNPK. Cell Insight 3, 100198 10.1016/j.cellin.2024.100198 39391005 PMC11462206

[26] Huang W.S., Xu F.M., Zeng Q.Z., Liu X.H., Gao X.J. and Liu L.X. (2017) ERK1/2-mediated cytoplasmic accumulation of hnRNPK antagonizes TRAIL-induced apoptosis through upregulation of XIAP in H1299 cells. Biomed. Environ. Sci. 30, 473–481 28756806 10.3967/bes2017.063

[27] Liepelt A., Mossanen J.C., Denecke B., Heymann F., De Santis R., Tacke F. et al. (2014) Translation control of TAK1 mRNA by hnRNP K modulates LPS-induced macrophage activation. RNA 20, 899–911 10.1261/rna.042788.113 24751651 PMC4024643

[28] Ostrowski J., Klimek-Tomczak K., Wyrwicz L.S., Mikula M., Schullery D.S. and Bomsztyk K. (2004) Heterogeneous nuclear ribonucleoprotein K enhances insulin-induced expression of mitochondrial UCP2 protein. J. Biol. Chem. 279, 54599–54609 10.1074/jbc.M406753200 15485813

[29] Puente L.G., Voisin S., Lee R.E. and Megeney L.A. (2006) Reconstructing the regulatory kinase pathways of myogenesis from phosphopeptide data. Mol. Cell. Proteomics 5, 2244–2251 10.1074/mcp.M600134-MCP200 16971385

[30] Nguyen T.M., Kabotyanski E.B., Reineke L.C., Shao J., Xiong F., Lee J.H. et al. (2020) The SINEB1 element in the long non-coding RNA Malat1 is necessary for TDP-43 proteostasis. Nucleic. Acids. Res. 48, 2621–2642 10.1093/nar/gkz1176 31863590 PMC7049706

[31] Poenisch M., Metz P., Blankenburg H., Ruggieri A., Lee J.Y., Rupp D. et al. (2015) Identification of HNRNPK as regulator of hepatitis C virus particle production. PLoS Pathog. 11, e1004573 10.1371/journal.ppat.1004573 25569684 PMC4287573

[32] Tanno M., Sakamoto J., Miura T., Shimamoto K. and Horio Y. (2007) Nucleocytoplasmic shuttling of the NAD^+^-dependent histone deacetylase SIRT1. J. Biol. Chem. 282, 6823–6832 10.1074/jbc.M609554200 17197703

[33] Haward F., Maslon M.M., Yeyati P.L., Bellora N., Hansen J.N., Aitken S. et al. (2021) Nucleo-cytoplasmic shuttling of splicing factor SRSF1 is required for development and cilia function. Elife 10, e65104 10.7554/eLife.65104 34338635 PMC8352595

[34] Mikula M., Dzwonek A., Karczmarski J., Rubel T., Dadlez M., Wyrwicz L.S. et al. (2006) Landscape of the hnRNP K protein–protein interactome. Proteomics 6, 2395–2406 10.1002/pmic.200500632 16518874

[35] Zhou W., Jie Q., Pan T., Shi J., Jiang T., Zhang Y. et al. (2023) Single-cell RNA binding protein regulatory network analyses reveal oncogenic HNRNPK-MYC signalling pathway in cancer. Commun. Biol. 6, 82 10.1038/s42003-023-04457-2 36681772 PMC9867709

[36] Peng W.Z., Liu J.X., Li C.F., Ma R. and Jie J.Z. (2019) hnRNPK promotes gastric tumorigenesis through regulating CD44E alternative splicing. Cancer Cell Int. 19, 335 10.1186/s12935-019-1020-x 31857793 PMC6909542

[37] Pintacuda G., Wei G., Roustan C., Kirmizitas B.A., Solcan N., Cerase A. et al. (2017) hnRNPK recruits PCGF3/5-PRC1 to the Xist RNA B-repeat to establish Polycomb-mediated chromosomal silencing. Mol. Cell 68, 955–969, 10.1016/j.molcel.2017.11.013 29220657 PMC5735038

[38] Navarro-Cobos M.J. and Brown C.J. (2025) Recruitment of chromatin remodelers by XIST B-repeat region is variably dependent on HNRNPK. Hum. Mol. Genet. 34, 229–238 10.1093/hmg/ddae173 39588742 PMC11792242

[39] Toki N., Takahashi H., Sharma H., Valentine M.N.Z., Rahman F.M., Zucchelli S. et al. (2020) SINEUP long non-coding RNA acts via PTBP1 and HNRNPK to promote translational initiation assemblies. Nucleic. Acids. Res. 48, 11626–11644 10.1093/nar/gkaa814 33130894 PMC7672464

[40] Ishii T., Igawa T., Hayakawa H., Fujita T., Sekiguchi M. and Nakabeppu Y. (2020) PCBP1 and PCBP2 both bind heavily oxidized RNA but cause opposing outcomes, suppressing or increasing apoptosis under oxidative conditions. J. Biol. Chem. 295, 12247–12261 10.1074/jbc.RA119.011870 32647012 PMC7443489

[41] Huelga S.C., Vu A.Q., Arnold J.D., Liang T.Y., Liu P.P., Yan B.Y. et al. (2012) Integrative genome-wide analysis reveals cooperative regulation of alternative splicing by hnRNP proteins. Cell Rep. 1, 167–178 10.1016/j.celrep.2012.02.001 22574288 PMC3345519

[42] Konig J., Zarnack K., Rot G., Curk T., Kayikci M., Zupan B. et al. (2010) iCLIP reveals the function of hnRNP particles in splicing at individual nucleotide resolution. Nat. Struct. Mol. Biol. 17, 909–915 10.1038/nsmb.1838 20601959 PMC3000544

[43] Wang Z., Chen J., Sun F., Zhao X., Dong Y., Yu S. et al. (2022) LncRNA CRLM1 inhibits apoptosis and promotes metastasis through transcriptional regulation cooperated with hnRNPK in colorectal cancer. Cell Biosci. 12, 120 10.1186/s13578-022-00849-9 35907898 PMC9338583

[44] Shin C.H., Ryu S. and Kim H.H. (2017) hnRNPK-regulated PTOV1-AS1 modulates heme oxygenase-1 expression via miR-1207-5p. BMB Rep 50, 220–225 10.5483/BMBRep.2017.50.4.024 28228215 PMC5437967

[45] Guo R., You X., Meng K., Sha R., Wang Z., Yuan N. et al. (2022) Single-cell RNA sequencing reveals heterogeneity of Myf5-derived cells and altered myogenic fate in the absence of SRSF2. Adv. Sci. (Weinh) 9, e2105775 10.1002/advs.202105775 35460187 PMC9218650

[46] Yue L., Wan R., Luan S., Zeng W. and Cheung T.H. (2020) Dek modulates global intron retention during muscle stem cells quiescence exit. Dev. Cell 53, 661–676, 10.1016/j.devcel.2020.05.006 32502396

[47] Xu Y., Xu H., Cheng X., Chen N., Wang Y., Huang Y. et al. (2024) Deletion of heterogeneous nuclear ribonucleoprotein K in satellite cells leads to inhibited skeletal muscle regeneration in mice. Genes Dis. 11, 101062 10.1016/j.gendis.2023.06.031 38510474 PMC10950818

[48] Xu Y., Qian H., Feng X., Xiong Y., Lei M., Ren Z. et al. (2012) Differential proteome and transcriptome analysis of porcine skeletal muscle during development. J. Proteomics 75, 2093–2108 10.1016/j.jprot.2012.01.013 22270015

[49] Chaze T., Meunier B., Chambon C., Jurie C. and Picard B. (2008) In vivo proteome dynamics during early bovine myogenesis. Proteomics 8, 4236–4248 10.1002/pmic.200701101 18924180

[50] Xu Y., Li R., Zhang K., Wu W., Wang S., Zhang P. et al. (2018) The multifunctional RNA-binding protein hnRNPK is critical for the proliferation and differentiation of myoblasts. BMB Rep. 51, 350–355 10.5483/BMBRep.2018.51.7.043 29898807 PMC6089871

[51] Zhang W., Zou M., Xiong X., Wei Y., Ke C., Li H. et al. (2024) Transcriptome analysis reveals the regulatory mechanism of myofiber development in male and female black Muscovy duck at different ages. Front Vet. Sci. 11, 1484102 10.3389/fvets.2024.1484102 39634756 PMC11614779

[52] Brinegar A.E., Xia Z., Loehr J.A., Li W., Rodney G.G. and Cooper T.A. (2017) Extensive alternative splicing transitions during postnatal skeletal muscle development are required for calcium handling functions. Elife 6, e27192 10.7554/eLife.27192 28826478 PMC5577920

[53] Xu Y., Wang Y., Cheng X., Zhang M., Chen N., Guo J. et al. (2025) The Paradox of hnRNPK: both absence and excess impair skeletal muscle function in mice. Skeletal Muscle 15, 20 10.1186/s13395-025-00393-3 40775642 PMC12329970

[54] Ubaida-Mohien C., Lyashkov A., Gonzalez-Freire M., Tharakan R., Shardell M., Moaddel R. et al. (2019) Discovery proteomics in aging human skeletal muscle finds change in spliceosome, immunity, proteostasis and mitochondria. Elife 8, e49874 10.7554/eLife.49874 31642809 PMC6810669

[55] Min B., Jeon K., Park J.S. and Kang Y.K. (2019) Demethylation and derepression of genomic retroelements in the skeletal muscles of aged mice. Aging Cell 18, e13042 10.1111/acel.13042 31560164 PMC6826136

[56] Lynch E.M., Robertson S., FitzGibbons C., Reilly M., Switalski C., Eckardt A. et al. (2021) Transcriptome analysis using patient iPSC-derived skeletal myocytes: Bet1L as a new molecule possibly linked to neuromuscular junction degeneration in ALS. Exp. Neurol. 345, 113815 10.1016/j.expneurol.2021.113815 34310943 PMC8429236

[57] Hitachi K., Nakatani M., Takasaki A., Ouchi Y., Uezumi A., Ageta H. et al. (2019) Myogenin promoter-associated lncRNA Myoparr is essential for myogenic differentiation. EMBO Rep. 20, e47468 10.15252/embr.201847468 30622218 PMC6399612

[58] Hitachi K., Kiyofuji Y., Nakatani M. and Tsuchida K. (2021) Myoparr-associated and -independent multiple roles of heterogeneous nuclear ribonucleoprotein K during skeletal muscle cell differentiation. Int. J. Mol. Sci. 23, 108 10.3390/ijms23010108 35008534 PMC8744952

[59] Hitachi K. and Tsuchida K. (2019) Data describing the effects of depletion of Myoparr, myogenin, Ddx17, and hnRNPK in differentiating C2C12 cells. Data Brief 25, 104172 10.1016/j.dib.2019.104172 31321265 PMC6612617

[60] Zhao Y., Zhou J., He L., Li Y., Yuan J., Sun K. et al. (2019) MyoD induced enhancer RNA interacts with hnRNPL to activate target gene transcription during myogenic differentiation. Nat. Commun. 10, 5787 10.1038/s41467-019-13598-0 31857580 PMC6923398

[61] Song Y., Liu Y., Chi R., Wang P., Di R., He X. et al. (2025) Study on the mechanism of LNC004268 regulating sheep myoblast proliferation and differentiation through hnRNPK-CNOT2 axis. Int. J. Biol. Macromol. 321, 146625 10.1016/j.ijbiomac.2025.146625 40774498

[62] Xu Y., Cheng X., Zhang M., Guo J., Ren K., Zhou K. et al. (2025) Unraveling the hnRNPK/miR-133a-3p/UCP2 axis: a novel regulatory circuit governing porcine skeletal muscle satellite cell fate. Cell. Signal. 136, 112160 10.1016/j.cellsig.2025.112160 41061811

[63] Ahn A., Kim J.J., Slusher A.L., Ying J.Y., Zhang E.Y. and Ludlow A.T. (2025) Impact of acute endurance exercise on alternative splicing in skeletal muscle. FASEB Bioadv. 7, e70024 10.1096/fba.2025-00007 40746864 PMC12312519

[64] Liu L., Luo C., Luo Y., Chen L., Liu Y., Wang Y. et al. (2018) MRPL33 and its splicing regulator hnRNPK are required for mitochondria function and implicated in tumor progression. Oncogene 37, 86–94 10.1038/onc.2017.314 28869607

[65] Yang J., Hung L.H., Licht T., Kostin S., Looso M., Khrameeva E. et al. (2014) RBM24 is a major regulator of muscle-specific alternative splicing. Dev. Cell 31, 87–99 10.1016/j.devcel.2014.08.025 25313962

[66] Kim H.J., Kim N.C., Wang Y.D., Scarborough E.A., Moore J., Diaz Z. et al. (2013) Mutations in prion-like domains in hnRNPA2B1 and hnRNPA1 cause multisystem proteinopathy and ALS. Nature 495, 467–473 10.1038/nature11922 23455423 PMC3756911

[67] Yokoi S., Ito T., Sahashi K., Nakatochi M., Nakamura R., Tohnai G. et al. (2022) The SYNGAP1 3′UTR variant in ALS patients causes aberrant SYNGAP1 splicing and dendritic spine loss by recruiting HNRNPK. J. Neurosci. 42, 8881–8896 10.1523/JNEUROSCI.0455-22.2022 36261283 PMC9698725

[68] Yao J., Tu Y., Shen C., Zhou Q., Xiao H., Jia D. et al. (2021) Nuclear import receptors and hnRNPK mediates nuclear import and stress granule localization of SIRLOIN. Cell. Mol. Life Sci. 78, 7617–7633 10.1007/s00018-021-03992-7 34689235 PMC11073023

[69] Lubelsky Y., Zuckerman B. and Ulitsky I. (2021) High-resolution mapping of function and protein binding in an RNA nuclear enrichment sequence. EMBO J. 40, e106357 10.15252/embj.2020106357 33938020 PMC8204871

[70] Cuvertino S., Martirosian E., Bhosale K., Cheng P., Garner T., Donaldson I.J. et al. (2025) Epigenome and transcriptome changes in KMT2D-related Kabuki syndrome type 1 iPSCs, neuronal progenitors and cortical neurons. PLos Genet. 21, e1011608 10.1371/journal.pgen.1011608 40971994 PMC12468740

[71] Choufani S., McNiven V., Cytrynbaum C., Jangjoo M., Adam M.P., Bjornsson H.T. et al. (2025) An HNRNPK-specific DNA methylation signature makes sense of missense variants and expands the phenotypic spectrum of Au-Kline syndrome. Am. J. Hum. Genet. 112, 1979 10.1016/j.ajhg.2025.07.001 40780051 PMC12414670

[72] Okamoto N. (2019) Okamoto syndrome has features overlapping with Au-Kline syndrome and is caused by HNRNPK mutation. Am. J. Med. Genet. A 179, 822–826 10.1002/ajmg.a.61079 30793470

[73] Song H., Feng X., Zhang H., Luo Y., Huang J., Lin M. et al. (2019) METTL3 and ALKBH5 oppositely regulate m^6^A modification of TFEB mRNA, which dictates the fate of hypoxia/reoxygenation-treated cardiomyocytes. Autophagy 15, 1419–1437 10.1080/15548627.2019.1586246 30870073 PMC6613905

[74] Doki T., Yamashita S., Wei F.Y., Hara K., Yamamoto T., Zhang Z. et al. (2019) Mitochondrial localization of PABPN1 in oculopharyngeal muscular dystrophy. Lab. Invest. 99, 1728–1740 10.1038/s41374-019-0243-8 30894671

[75] Lee E.J., Charles J.F., Sinha I. and Neppl R.L. (2024) Loss of HNRNPU in skeletal muscle increases intramuscular infiltration of Ly6C positive cells, leading to muscle atrophy through activation of NF-kappaB signaling. Adv. Biol. (Weinh) 8, e2400152 10.1002/adbi.202400152 38797891

[76] Xiong J., Le Y Rao, Y, Zhou L., Hu Y., Guo S. et al. (2021) RANKL mediates muscle atrophy and dysfunction in a cigarette smoke-induced model of chronic obstructive pulmonary disease. Am. J. Respir. Cell Mol. Biol. 64, 617–628 10.1165/rcmb.2020-0449OC 33689672

[77] Abrigo J., Elorza A.A., Riedel C.A., Vilos C., Simon F., Cabrera D. et al. (2018) Role of oxidative stress as key regulator of muscle wasting during cachexia. Oxid. Med. Cell Longev. 2018, 2063179 10.1155/2018/2063179 29785242 PMC5896211

[78] Graham C.D., Rose M.R., Grunfeld E.A., Kyle S.D. and Weinman J. (2011) A systematic review of quality of life in adults with muscle disease. J. Neurol. 258, 1581–1592 10.1007/s00415-011-6062-5 21597956

[79] Wang Z., Qiu H., He J., Liu L., Xue W., Fox A. et al. (2020) The emerging roles of hnRNPK. J. Cell. Physiol. 235, 1995–2008 10.1002/jcp.29186 31538344

[80] Braems E., Bercier V., Van Schoor E., Heeren K., Beckers J., Fumagalli L. et al. (2022) HNRNPK alleviates RNA toxicity by counteracting DNA damage in C9orf72 ALS. Acta Neuropathol. 144, 465–488 10.1007/s00401-022-02471-y 35895140 PMC9381635

[81] Purice M.D. and Taylor J.P. (2018) Linking hnRNP function to ALS and FTD pathology. Front Neurosci. 12, 326 10.3389/fnins.2018.00326 29867335 PMC5962818

[82] Wang S. and Sun S. (2023) Translation dysregulation in neurodegenerative diseases: a focus on ALS. Mol. Neurodegener. 18, 58 10.1186/s13024-023-00642-3 37626421 PMC10464328

[83] Xu Q., Wang X., Hu J., Wang Y., Lu S., Xiong J. et al. (2025) Overexpression of hnRNPK and inhibition of cytoplasmic translocation ameliorate lipid disorder in doxorubicin-induced cardiomyopathy via PINK1/Parkin-mediated mitophagy. Free Radic. Biol. Med. 231, 94–108 10.1016/j.freeradbiomed.2025.02.021 39984063

[84] Zhang J., Liu X., Yin C. and Zong S. (2022) hnRNPK/Beclin1 signaling regulates autophagy to promote imatinib resistance in Philadelphia chromosome-positive acute lymphoblastic leukemia cells. Exp. Hematol. 108, 46–54 10.1016/j.exphem.2022.01.004 35038545

[85] Yanay N., Rabie M. and Nevo Y. (2020) Impaired regeneration in dystrophic muscle—new target for therapy. Front Mol. Neurosci. 13, 69 32523512 10.3389/fnmol.2020.00069 PMC7261890

[86] Stemmerik M.G., Tasca G., Gilhus N.E., Servais L., Vicino A., Maggi L. et al. (2025) Biological biomarkers in muscle diseases relevant for follow-up and evaluation of treatment. Brain 148, 363–375 10.1093/brain/awae323 39397743

[87] Ohlendieck K. (2013) Proteomic identification of biomarkers of skeletal muscle disorders. Biomark Med. 7, 169–186 10.2217/bmm.12.96 23387498

[88] Guo L., Han M., Xu J., Zhou W., Shi H., Chen S. et al. (2025) snRNA-seq and spatial transcriptome reveal cell–cell crosstalk mediated metabolic regulation in porcine skeletal muscle. J. Cachexia Sarcopenia Muscle 16, e13752 10.1002/jcsm.13752 40079370 PMC11904818

[89] Harley J. and Patani R. (2020) Stress-specific spatiotemporal responses of RNA-binding proteins in human stem-cell–derived motor neurons. Int. J. Mol. Sci. 21, 8346 10.3390/ijms21218346 33172210 PMC7664327

